# Plasma Levels of Interleukins 36α, 36β, and 37 in Patients with Psoriasis and Their Correlation with Disease Activity Parameters

**DOI:** 10.3390/jcm11185254

**Published:** 2022-09-06

**Authors:** Sylwia Słucznowska-Głabowska, Weronika Jaworska, Marzena Staniszewska, Marta Tkacz, Krzysztof Safranow, Karolina Łuczkowska, Edyta Zagrodnik, Iwona Stecewicz, Bogusław Machaliński, Andrzej Pawlik

**Affiliations:** 1 Department of Physiology, Pomeranian Medical University, 70-111 Szczecin, Poland; 2 Department of Biochemistry and Medical Chemistry, Pomeranian Medical University, 70-111 Szczecin, Poland; 3 Department of General Pathology, Pomeranian Medical University, 70-111 Szczecin, Poland; 4 Department of Anestesiology, Pomeranian Medical University, 70-111 Szczecin, Poland; 5 Department of Pediatrics, Pomeranian Medical University, 70-111 Szczecin, Poland

**Keywords:** psoriasis, interleukins, inflammation

## Abstract

Psoriasis is a chronic, proliferative, inflammatory skin disease characterised by skin lesions and systemic symptoms. Numerous cytokines are produced in psoriasis as a result of inflammation. The aim of this study was to examine the plasma concentrations of IL-36α, IL-36β, and IL-37 in psoriasis and their correlations with disease activity parameters. This study recruited 84 individuals, 53 with plaque-type psoriasis and 31 healthy controls. The plaque type of psoriasis is the most common type and is typically characterized by circular-to-oval red plaques distributed over body surfaces of the extremities and scalp. In patients with psoriasis, we observed statistically significantly decreased plasma concentrations of IL-36β and IL-37. The concentrations of IL-36α were increased in comparison with control group. The plasma concentrations of IL-36α and IL-36β were statistically significantly correlated with all tested parameters of disease activity: the Psoriasis Activity Severity Index, Dermatology Life Quality Index, and Body Surface Area Index. There were no statistically significant correlations between plasma levels of IL-37 and the tested parameters of disease activity. These results indicate a role of IL36α, IL-36β, and IL-37 in the pathogenesis of psoriasis.

## 1. Introduction

Psoriasis, the most common chronic, proliferative, and inflammatory skin disease, is characterized by raised, red, scaly plaques and possible systemic manifestations. As a chronic disease with periods of exacerbation and remission, it has a significantly greater impact on the quality of patients’ physical and emotional lives than other serious diseases. The most common variant of this disease, affecting 85–90% of patients, is plaque psoriasis [1]. In addition to isolated skin lesions, 25% of patients with psoriasis and joint lesions are diagnosed with psoriatic arthritis [2]. Moreover, psoriasis can be associated with other diseases, such as cardiological, metabolic, gastrointestinal, kidney, and mental disorders [3,4,5,6]. 

The prevalence of psoriasis is in about 2–3% of the world’s population and varies between ethnic groups. Higher rates of illness have been observed in Western countries: about 2.2% in the UK and 3.15% in the United States but less than 0.5% in Asia. This inequality may result from differences in genetic, ethnic, environmental, and climate factors [7]. Equally common in women and men, psoriasis is a disease with a genetic basis and multigenetic inheritance. Many factors—environmental, hormonal, and immunological—play a role in the development of psoriasis. However, the influence of genetic conditions and a multigene background is emphasized [8,9,10]. 

Immunological, even autoimmune, phenomena and related activation of keratinocytes and T lymphocytes as well as a reduction in the number of antigen-presenting Langerhans cells that migrate to the skin as part of inflammatory infiltration all play key roles in the etiopathogenesis of psoriasis. In these cases, keratinocytes produce many cytokines that are responsible for the expression of cell membrane proteins, such as HLA-DR and ICAM-1 [11]. In epidermal damage, neutrophils and lymphocytes are observed to migrate to the damaged site following cytokine stimulation. Very often, the first psoriatic lesions are associated with the initiation of immunological processes by superantigens or antigens that do not induce the synthesis of antibodies directed against them. They appear most often after bacterial or viral infections and show some homology and cross-reactions with keratinocyte antigens, which explains autoimmunity [12]. Superantigens are presented by keratinocytes expressing MCH II antigens. As a result of such presentation, T cells, especially T helper cells (Th17), are activated, and several proinflammatory cytokines are produced. Thus, the inflammatory response in psoriasis is principally driven by T cells, especially T helper cells (Th17), and mediated by various cytokines [13] derived not only from T cells but also from keratinocytes and other immune cells. These processes are regulated by numerous pro-inflammatory and anti-inflammatory cytokines

IL-36 is a member of the IL-1 family of cytokines and consists of IL-36α, β, γ, and IL-36Ra. The genes encoding IL-36 are located on chromosome 2q13. These cytokines are synthesized mainly by keratinocytes, endothelial cells, and various cells of the immune system [14]. IL-36α, β, γ, and IL-36Ra are synthesized as inactive and undergo proteolytic cleavage for activation. This process involves proteases secreted from neutrophils or lymphocytes, such as cathepsin G, elastase, and proteinase-3. IL-36 plays an important role in immune processes by stimulating both innate and adaptive immune responses [15]. It has been shown that IL-36 can stimulate macrophages and T lymphocytes to secrete pro-inflammatory cytokines involved in the pathogenesis of psoriasis [15]. Previous studies have confirmed the important role of IL-36 in various immune-related diseases, including inflammatory and autoimmune diseases [16]. These findings suggest that activation of IL-36 on immune or epithelial cells can stimulate innate and adaptive immune cells, such as dendritic cells and naive CD4+ T cells, to stimulate host responses against various antigens. These processes underlie the development of psoriasis [14].

IL-37 is a cytokine of the IL-1 family with anti-inflammatory and immunosuppressive activity [17]. IL-37 has been detected in various cells and tissues, such as skin and intestine, as well as in immune cells, including monocytes, macrophages, dendritic cells, T and B lymphocytes, and plasmatic cells [18]. Pro-inflammatory factors have been shown to increase the expression of IL-37. These findings suggest that IL-37 may play a key role in maintaining the homeostatic balance of the immune system, probably particularly in the immune barrier. Studies have shown that IL-37 plays a role in the development of and pathogenesis of inflammatory diseases, autoimmune metabolic disorders, and cancer, making it a potential target for therapy [19]. IL-37 expression has been confirmed in keratocytes, which may suggest its role in keratinocyte differentiation and keratinization. In the skin, IL-37 has been shown to inhibit the expression of various pro-inflammatory mediators, including IL-6 and CXCL8, which are involved in the pathogenesis of psoriasis [18].

The aim of this study was to examine the plasma concentrations of IL-36α, IL-36β, and IL-37 in psoriasis and their correlation with disease activity parameters.

## 2. Materials and Methods

### 2.1. Patients

This study recruited 84 individuals, 53 with plaque-type and 31 healthy controls. The plaque type of psoriasis is the most common type and is most typically characterized by circular-to-oval red plaques distributed over body surfaces of the extremities and scalp. Patients were recruited from the Dermatology Clinic of Pomeranian University in Szczecin, Poland. Each participant was thoroughly examined by a dermatologist, who classified their psoriasis according to the International Classification of Diseases, Tenth Revision [20]. The diagnosis was determined by clinical features, and the disease activity was measured using the Psoriasis Activity Severity Index (PASI), Dermatology Life Quality Index (DLQI) and Body Surface Area (BSA) index. These indexes were evaluated by the same investigator. 

Currently, psoriasis activity is assessed by the PASI (Psoriasis Area Severity Index), BSA (Body Surface Area) and DLQI (Dermatology Life Quality Index) indices [20]. One of the most common indices used in clinical practice to assess the severity of psoriasis is the PASI scale. It considers the presence of erythema, lesion thickness, and scale build-up, as well as the percentage of the skin surface occupied in four locations: the head, trunk, and upper and lower extremities. BSA is an index that assesses the percentage of the skin area affected by psoriatic lesions. The DLQI is one of the most widely used scales to assess the impact of the presence of psoriatic lesions on patients’ quality of life and the extent to which treatment improves it.

The included patients were of both sexes, aged from 18 to 70 years and had received only topical treatment. The exclusion criteria were systemic treatment of psoriasis (biologic drugs, immunosuppressive drugs, and other antipsoriatic drugs used orally) and thyroid, adrenal, renal, hepatic, gastrointestinal, oncological, or another autoimmune disease. Information on lifestyle factors and medical history was obtained at the clinical evaluation. A detailed history included disease duration, age at disease diagnosis, familial history of psoriasis, and lifestyle factors, such as tobacco smoking and alcohol consumption. Venous blood samples (5 mL) were collected using vacutainer tubes. This study was conducted according to the guidelines laid down in the Declaration of Helsinki, and all procedures involving human subjects/patients were approved by the Ethical Committee of the Pomeranian University in Szczecin (Poland), number KB-0012/105/17.

### 2.2. Assessment of IL-36α, IL-36β, and IL-37 Concentrations in the Plasma of Patients with Psoriasis and Control Subjects

The plasma concentrations of IL-36α, IL-36β, and IL-37 were tested in each sample using a magnetic bead-based multiplex assay according to the manufacturer’s procedure (Luminex Assay R&D, Minneapolis, MN, USA).

### 2.3. Statistical Analysis

Since the distributions of quantitative variables differed significantly from the normal distribution (Shapiro–Wilk test), non-parametric tests were used. Values were compared between groups with a Mann–Whitney test, and correlations within groups were assessed with the Spearman rank correlation coefficient. A *p*-value of <0.05 was considered to indicate a statistically significant result.

## 3. Results

### 3.1. Analysis of IL-36α, IL-36β, and IL-37 Concentrations in the Plasma of Patients with Psoriasis and Control Subjects

The clinical characteristics of patients and control group are shown in Table 1. In both groups, we observed large inter-individual differences in plasma concentrations of studied cytokines, which resulted in large standard deviations in the obtained results. There were no patients with psoriatic arthritis in the study group.

**Figure 1:**
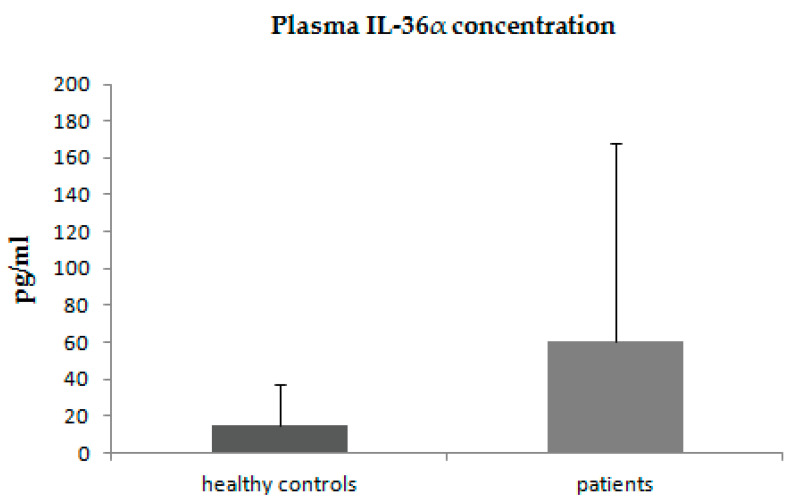
Plasma concentrations of IL-36α in patients with psoriasis and control group.

The levels of IL-36α were increased in patients with psoriasis in comparison with the control group; however, due to large inter-individual differences between patients, these differences did not reach statistical significance (Figure 1). 

**Figure 2:**
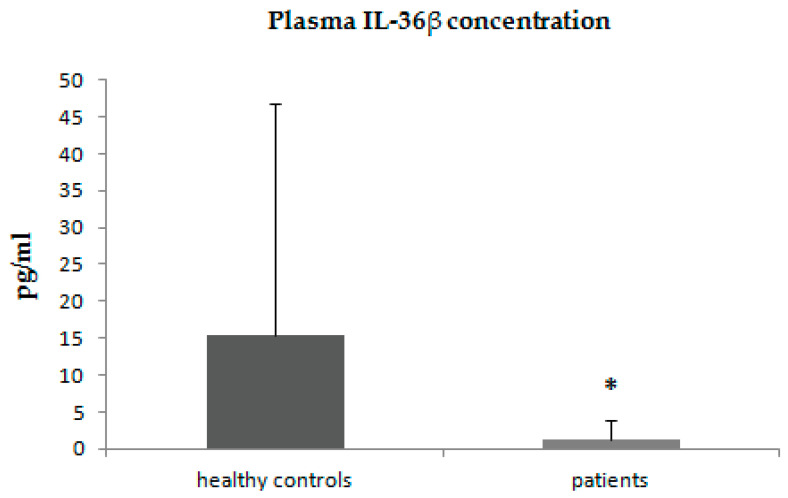
Plasma concentrations of IL-36β in patients with psoriasis and control group. * *p* < 0.001.

In patients with psoriasis, we observed statistically significantly decreased plasma concentrations of IL-36β (Figure 2).

**Figure 3:**
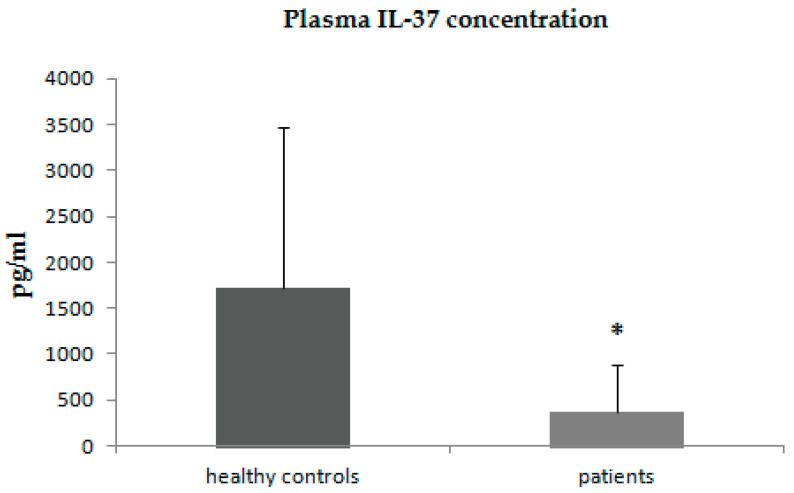
Plasma concentrations of IL-37 in patients with psoriasis and control group. * *p* < 0.001.

The concentrations of IL-37 were statistically significantly lower in patients with psoriasis in comparison with control subjects (Figure 3).

Additionally, we compared the plasma concentrations of IL-36α, IL-36β, and IL-37 between men and women with psoriasis. These differences were not statistically significant (*p* = 0.45, *p* = 0.15, and *p* = 0.1, respectively).

We also compared the plasma concentrations of IL-36α, IL-36β, and IL-37 between smoking and non-smoking patients. These differences were not statistically significant (*p* = 0.37, *p* = 0.87, and *p* = 0.6, respectively). 

### 3.2. Correlations between Plasma Concentrations of IL-36α, IL-36β, and IL-37 and Clinical Parameters in Patients with Psoriasis

The next point of our study was to examine the correlations between plasma concentrations of IL-36α, IL-36β, and IL-37 and clinical parameters.

There were no statistically significant correlations between plasma concentrations of IL-36α, IL-36β, and IL-37 and age of patients as well as age of disease onset (Table 2).

The plasma concentrations of IL-36α were statistically significantly correlated with all parameters of disease activity tested. In addition, plasma concentrations of IL-36α were correlated with those of hemoglobin as well as aspartate aminotransferase (AST) and alanine aminotransferase (ALT) values (Table 2).

The plasma concentrations of IL-36β were statistically significantly correlated with all parameters of disease activity: DLQI, PASI, and BSA. Additionally, plasma concentrations of IL-36β were correlated with aspartate aminotransferase and alanine aminotransferase values (Table 2).

There were no statistically significant correlations between plasma levels of IL-37 and any of the tested parameters of disease activity or other studied clinical parameters (Table 2).

## 4. Discussion

In this study, we examined the plasma concentrations of IL-36α, IL-36β, and IL-37 in patients with psoriasis. In addition, we correlated the plasma concentrations of IL-36α, IL-36β, and IL-37 with disease activity parameters and other selected clinical parameters. In patients with psoriasis, we observed statistically significantly decreased plasma concentrations of IL-36β and IL-37, whereas plasma concentrations of IL-36α were increased. Plasma concentrations of IL-36α and IL-36β were significantly correlated with disease activity parameters (DLQI, PASI, and BSA), whereas there were no statistically significant correlations between IL-37 plasma levels and DLQI, PASI, and BSA values. 

We did not detect differences in plasma concentrations of IL-36α, IL-36β, and IL-37 between men and women with psoriasis and smoking and non-smoking patients. Hayran et al. found significantly higher levels of IL-36α, IL-36β in patients with hidradenitis suppurativa in smokers than in non-smokers [21]. 

Of interest is the fact of elevated IL-36α and reduced IL-36β levels in patients with psoriasis. Despite this, IL-36β concentrations correlated statistically significantly with disease activity parameters. Psoriasis is a disease in which pathogenesis and a number of pro-inflammatory and anti-inflammatory mediators are involved. In addition, IL-36 stimulates the synthesis of many other mediators such as cytokines and chemokines [15,16]. It is likely that some of them can inhibit IL-36β synthesis. However, a thorough understanding of this process requires further research. 

Psoriasis is a chronic inflammatory skin disease. A search is currently ongoing for factors triggering the onset of this disease, as well as those influencing its course and activity. Previous studies have shown that several cytokines are involved in the pathogenesis of psoriasis, both leading to the onset of disease symptoms and exacerbating them [22]. 

The results of our study suggest that all evaluated cytokines are involved in the pathogenesis of psoriasis. Moreover, plasma levels of IL-36α and IL-36β are correlated with the disease activity parameters DLQI, PASI, and BSA. We also observed the correlations between IL-36α and plasma concentrations and the values of AST, ALT, hemoglobin, and creatinine. It has been shown that IL-36 signaling contributes to the pathogenesis of renal tubulointerstitial lesions through the activation of the NLRP3 inflammasome and IL-23/IL-17 axis. IL-36 can cause changes in interstitial tubules and thus affect creatinine levels [23].

It has also shown that IL-36 is involved in pathogenesis of several inflammatory diseases, such as obesity, atherosclerosis, as well as liver diseases [24,25]. Through the modulating innate lymphoid immunity, IL-36 may enhance the inflammation in the liver [26]. It is likely that, through these mechanisms, IL-36 can affect AST and ALT values. In addition, IL-36 has been shown to increase the expression of other cytokines and pro-inflammatory mediators, such as IL-6, which can also increase AST and ALT activity [15]. By increasing the expression of many other mediators and growth factors, IL-36 can probably also affect hemoglobin synthesis.

Several studies have examined the role of IL-36 and IL-37 in the pathogenesis of psoriasis; however, the influence of these cytokines on the clinical course of psoriasis is not fully understood. IL-36 consists of four isoforms: IL-36α, IL-36β, IL-36γ, and an IL-36 receptor antagonist (IL-36 Ra). IL-36α and IL-36γ are produced by keratinocytes, endothelia cells, macrophages, dendritic cells, and Langerhans cells, whereas IL-36β is produced by endothelial cells [15,27]. It has been demonstrated that the expression of IL-36 in keratinocytes is induced by TNF, IL-17, and IL-22 [14]. 

To date, the expression of IL-36 isoforms in the skin has been studied but not their serum levels. Previous studies have examined the serum concentration of total IL-36 without determining its different isotypes. In our study, we assessed serum levels of IL-36α and IL-36β isoforms in patients with psoriasis.

Boutet et al. have shown that patients with psoriasis had significantly higher skin mRNA levels of IL-36α and IL-36γ and IL-36Ra, whereas IL-36β was similar as in the control group [28]. The studies have also shown that psoriatic lesions express significant levels of IL-36α, and this is correlated with increased production of Th1 and Th17 cytokines, including: IL-6, IL-8, IL-17A, IL-22, and TNFα [29]. Sehat et al. have shown that serum levels of total IL-36 in patients with psoriasis vulgaris were significantly higher than those in healthy controls and positively correlated with disease activity (PASI score) [30]. In our study, we have indicated increased levels of IL-36α and decreased levels of IL-36β in patients with psoriasis. Both IL-36α and IL-36β levels correlated significantly with disease activity parameters DLQI, PASI, and BSA.

IL-36 is a pro-inflammatory cytokine that occurs physiologically in the skin. Increased expression of IL-36 has been found in the skin of patients with psoriasis; in addition, inflammatory factors, such as TNF and LPS, have been shown to increase IL-36 expression [31]. Psoriatic skin lesions are characterized by hyperproliferation and altered keratinocyte differentiation. IL-36 inhibits keratinocyte differentiation and induces a pro-inflammatory phenotype and the development of skin lesions [32,33].

IL-36 has been shown to increase the expression of other pro-inflammatory mediators, such as IL-1, IL-12, IL-23, IL-6, TNF, CXCL1, and GM-CSF [34,35]. This cytokine also increases the activity and proliferation of lymphocytes, which participate actively in the pathogenesis of psoriasis. IL-36 also activates the endothelium and enhances the expression of adhesive particles (VCAM-1 and ICAM-1), which increase inflammation [36]. Additionally, IL-36 increases the expression of Th17 cytokines through activation of Th17 cells [37]. The role of IL-36 has also been confirmed in experimental models: IL-36R knockout mice are protected from imiquimod-induced skin lesions observed in wild-type mice, whereas knockout of IL-36Ra increases the occurrence of skin lesions [38]. 

IL-36 plays an important role in signaling between epithelial cells, dendritic cells, and neutrophils, which are responsible for the initiation, continuation, and exacerbation of inflammation [14,15]. It has also been shown that treatment of psoriasis reduces the expression of IL-36, which is correlated with a reduction in the activity of disease parameters [39].

IL-36 has been shown to be involved in signaling pathways in skin cells, particularly in endothelial cells and keratinocytes [14,15,40,41]. In keratinocytes, IL-36 increases the release of pro-inflammatory cytokines and chemokines, increases levels of AMP and growth factors, and affects keratinization [40,41]. IL-36 also increases the expression of adhesion molecules and activates macrophages and neutrophils, in particular, in clinical forms of psoriasis in which neutrophils play a major role, such as generalized pustular psoriasis and palmoplantar pustular psoriasis. Patients with generalized pustular psoriasis and palmoplantar pustular psoriasis have been shown to have mutations in the IL-36 gene region [42]. Interestingly, a higher prevalence of mutations in the IL-36 gene was found in generalized pustular psoriasis compared to the palmoplantar pustular psoriasis patient population and has also been linked to an earlier age of onset of pustular psoriasis [42].

Recent studies have shown that blocking the IL-36 pathway reduces clinical, histological, and immunohistochemical changes in the psoriatic skin, including neutrophil and chemokine infiltration [31,43]. IL-36 thus appears to be a promising target for psoriasis therapy. The effect of blocking IL-36 on the course of psoriasis has been extensively studied in several experimental models. Mice deficient in IL-36 or treated with IL-36-blocking antibodies were protected from induced dermatitis [43]. Antibodies antagonistic to IL-36 also inhibited the inflammatory response in other models of psoriatic dermatitis by reducing leukocyte infiltration and acanthosis [44,45]. In addition, it was found that blocking IL-36 reduces the formation of psoriatic lesions and the expression of genes associated with the development of psoriasis. However, it has been shown that mice lacking the IL-36Ra antagonist are at increased risk of developing psoriasis [46,47]. 

Spesolimab and imsidolimab are two IL-36 receptor inhibitors that have undergone phase 1 and phase 2 trials for the treatment of psoriasis [48]. These drugs have been shown to have a good efficacy and safety profile in psoriasis therapy, including rapid control of exacerbations. Therapy with these drugs did not cause significant side effects requiring treatment discontinuation. The effectiveness of these drugs has been confirmed especially in pustular psoriasis [15,48]. Overall, blocking IL-36 may be a promising therapeutic strategy for the treatment of psoriasis, especially pustular forms, in which hyperactivation of the IL-36 axis contributes to recruitment and activation of neutrophils.

IL-37 is an anti-inflammatory cytokine whose expression has been confirmed in keratinocytes [49]. IL-37 has been shown to exert anti-inflammatory effects by inhibiting IL-6 and IL-8, among others [50]. IL-37 also inhibits neutrophil migration and infiltration in inflammatory skin diseases [51,52]. To date, IL-37 has not been extensively studied in psoriasis patients, although its role in inhibiting skin inflammation has been confirmed in human and animal models of psoriasis. 

Previous studies suggest a role for IL-37 in the pathogenesis of psoriasis, but the findings are inconsistent. Both elevated and reduced IL-37 expression has been demonstrated in various forms of psoriasis [50,51,52]. Rønholt et al. demonstrated that IL-37 is down-regulated in human lesional psoriasis skin [51]. Sehat et al. have shown that gene expression levels of IL-37 in patients with psoriasis were higher than those in healthy controls but were not correlated with disease activity parameters [30]. In other studies, reduced expression of IL-37 has been observed in psoriatic skin lesions compared to unaffected skin [52]. Additionally, other studies suggest that IL-37 has anti-inflammatory effects on psoriasis. Teng et al. have shown that IL-37 ameliorates the inflammatory process in psoriasis by suppressing pro-inflammatory cytokine production [50]. In our study, we observed reduced serum IL-37 levels in patients with psoriasis that did not correlate with disease activity parameters. 

Currently, there is a growing search for new therapeutic targets in psoriasis and biomarkers to help diagnose the disease, assess its activity and progression, as well as the effectiveness of the therapy used. Psoriasis is a chronic disease, but so far in clinical practice, we have very few biomarkers to monitor its course and activity. It has been shown that extracellular vesicles secreted by cells and present in biological fluids can play an important role in the diagnosis and monitoring of many diseases, including psoriasis [53]. Changes in the lipid profile in extracellular vesicles have been demonstrated in patients with psoriasis, suggesting that determining lipid changes in circulating extracellular vesicles may be helpful in the diagnosis and evaluation of drug response in patients with psoriasis [53].

## 5. Conclusions

The results of our study indicated increased plasma levels of IL36α and decreased plasma levels of IL-36β and IL-37 in patients with psoriasis. In addition, plasma levels of IL-36α and IL-36β were significantly correlated with the disease activity parameters DLQI, PASI, and BSA. These results indicate the role of IL36α, IL-36β, and IL-37 in the pathogenesis of psoriasis. 

## Figures and Tables

**Table 1:** Clinical characteristics of patients and control group.

Parameters	Patients with Psoriasis n-53	Control Group n-31
Gender M/F	29/24	17/14
Age [years]	49.7 ± 17.6	48.5 ± 14.2
Age of disease onset [years]	34.03 ± 20.08	-
Smoking	19	12
DLQI	11.7 ± 8.19	-
PASI	11.28 ± 10.87	-
BSA	21.33 ± 21.18	-

DLQI—Dermatology Life Quality Index, PASI—Psoriasis Area Severity Index, BSA—Body Surface Area.

**Table 2:** Correlations between IL-36α, IL-36β, and IL-37 plasma concentrations and clinical parameters.

	IL-36α		IL-36β		IL-37	
Parameter	Rs	*p*	Rs	*p*	Rs	*p*
Age	−0.0195	0.88	−0.1922	0.16	−0.0999	0.47
Age of disease onset	−0.1078	0.44	−0.1384	0.32	−0.1462	0.3
DLQI	0.2849	0.04	0.5389	<0.001	0.1767	0.21
PASI	0.4268	0.001	0.4852	<0.001	0.1647	0.23
BSA	0.3606	0.007	0.4829	<0.001	0.1772	0.2
Erythrocytes	0.1987	0.36	0.2243	0.3	0.1399	0.52
Hemoglobin	0.439	0.03	0.3959	0.06	0.1483	0.49
Leukocytes	−0.3419	0.11	−0.4394	0.03	0.1126	0.6
ESR	−0.1616	0.46	−0.068	0.75	0.0373	0.86
CRP	−0.1302	0.55	−0.186	0.39	−0.0908	0.68
AST	0.4114	0.05	0.7832	<0.001	0.3213	0.13
ALT	0.4519	0.03	0.4003	0.05	0.1287	0.55
Creatinin	0.4584	0.03	0.2678	0.22	0.3122	0.15

DLQI—Dermatology Life Quality Index, PASI—Psoriasis Area Severity Index, BSA—Body Surface Area, ESR—erythrocyte sedimentation rate, CRP—C-reactive protein, AST—aspartate aminotransferase, ALT—alanine aminotransferase.

## Data Availability

Not applicable.
